# A Unified Lattice Boltzmann Model for Fourth Order Partial Differential Equations with Variable Coefficients

**DOI:** 10.3390/e24091176

**Published:** 2022-08-23

**Authors:** Wei Yang, Chunguang Li

**Affiliations:** School of Mathematics and Information Science, North Minzu University, Yinchuan 750021, China

**Keywords:** unified lattice Boltzmann model, partial differential equations, variable coefficients, numerical simulations, soliton solutions

## Abstract

In this work, a unified lattice Boltzmann model is proposed for the fourth order partial differential equation with time-dependent variable coefficients, which has the form $u_{t}+\alpha \left(t\right){\left(p_{1}\left(u\right)\right)}_{x}+\beta \left(t\right){\left(p_{2}\left(u\right)\right)}_{xx}+\gamma \left(t\right){\left(p_{3}\left(u\right)\right)}_{xxx}+\eta \left(t\right){\left(p_{4}\left(u\right)\right)}_{xxxx}=0$. A compensation function is added to the evolution equation to recover the macroscopic equation. Applying Chapman-Enskog expansion and the Taylor expansion method, we recover the macroscopic equation correctly. Through analyzing the error, our model reaches second-order accuracy in time. A series of constant-coefficient and variable-coefficient partial differential equations are successfully simulated, which tests the effectiveness and stability of the present model.

## 1. Introduction

The analytical solution, also known as the exact solution, can accurately solve the partial differential equations( PDEs) [1,2,3,4], but it is quite difficult for us to obtain analytical solutions. The analytical solution may even be limited to some special types of PDEs. In such a context, it is very necessary and significant to study the numerical solution of PDEs. Compared with traditional numerical methods such as the finite difference method, finite volume method, finite element method, spectral method and so on, lattice Boltzmann method (LBM) is a novel numerical method with unique advantages, such as simple programming, easy treatment of boundary conditions and fit for parallel computing.

As a mesoscopic numerical simulation method, LBM has made some progress in the past 30 years [5,6]. With time and space made discrete, LBM is macroscopically discrete. However, LBM is microscopically continuous, which satisfies the conservation of mass, momentum conservation and energy conservation. The macroscopic equation can be correctly recovered by LBM. LBM attracts more and more experts and scholars’ attention internationally. Researchers mainly use LBM to simulate fluid flow [7,8] and solve PDEs [9,10,11,12,13,14].

Lai and Ma [9] presented a lattice Boltzmann (LB) model for fourth order generalized Kuramoto-Sivashinsky (GKS) equation, in which an amending function assumed to be second order of time step is applied to recover the GKS equation correctly. Hu and their collaborators [10] developed a LB model to solve a generalized Gardner equation with time-dependent variable coefficients (TDVCs) by means of adding a compensation function to the evolution equation. In order to solve a class of PDEs with the order up to six, Chai  et al. [11] introduced some suitable auxiliary moments to study a general LB model. Lan et al. [12] investigated a general propagation LB model and successfully simulated KdV-Burgers equation with TDVCs through adjusting the propagation step. Following the idea in the work of Chai and his collaborators, Qiao et al. [13] recently proposed a novel LB model with an auxiliary source distribution function to solve the general fourth order PDEs. In our previous work [14], a general propagation lattice Boltzmann model was developed to solve Boussinesq equations by selecting the appropriate parameters that affect the propagation process. In this work, we develop a unified lattice Boltzmann model for fourth order PDEs with TDVCs, so as to avoid the need to construct different models for different PDEs, including constant-coefficient PDEs and variable-coefficient PDEs. Furthermore, the equilibrium distribution functions and the compensation functions have a unified form for different PDEs according to our unified model. Based on our model and algorithm, the constant-coefficient KS equation, constant-coefficient GKS equation and variable-coefficient KdV equation, are successfully solved numerically.

We organize the paper as follows: a unified lattice Boltzmann model for fourth order partial differential equations with variable coefficients is proposed in Section 2, where we give a detailed derivation and analysis process of important equations, such as the Chapman-Enskog analysis, the equilibrium distribution functions, the compensation functions and so on. In Section 3, the algorithm for the present model and numerical results are shown. In the end, Section 4 is a summary of this work.

## 2. A Unified Lattice Boltzmann Model for Fourth Order Partial Differential Equations with Variable Coefficients

A unified LB model for fourth order PDEs with TDVCs, i.e., Equation (1) is investigated,

(1) $$\frac{\partial u}{\partial t}+\alpha \left(t\right)\frac{\partial }{\partial x}p_{1}\left(u\right)+\beta \left(t\right)\frac{\partial^{2}}{\partial x^{2}}p_{2}\left(u\right)+\gamma \left(t\right)\frac{\partial^{3}}{\partial x^{3}}p_{3}\left(u\right)+\eta \left(t\right)\frac{\partial^{4}}{\partial x^{4}}p_{4}\left(u\right)=0.$$

In Equation (1), x,t represent spatial position and time, respectively, u=u(x,t) is an unknown scalar function. It should be noticed that unlike previous work, the coefficients α(t),β(t),γ(t),η(t) in front of the partial derivative of space are functions of time, i.e., time-dependent variable coefficients, and $P_{i}\left(u\right)\left(i=1,2,\ldots ,4\right)$ is a polynomial function of *u*, such as $p_{1}\left(u\right)=u^{2}$ and so on.

### 2.1. A Unified Lattice Boltzmann Model for Fourth Order Partial Differential Equations with Variable Coefficients

As for Equation (1), we introduce the discrete velocity lattice Boltzmann equation with Bhatnagar-Gross-Krook (BGK) collision operator, which satisfies this form:

(2) $$f_{k}\left(x+\alpha_{k}\Delta t,t+\Delta t\right)-f_{k}\left(x,t\right)=\frac{\Delta t[f_{k}^{eq}\left(x,t\right)-f_{k}\left(x,t\right)]}{\tau_{0}}+\Delta tg_{k}\left(x,t\right),$$

where $f_{k}\left(x,t\right)$ and $f_{k}^{eq}\left(x,t\right)\left(k=0,1,\ldots ,n-1\right)$ are the local particle distribution function and the local equilibrium distribution function, respectively, and $\tau_{0}$ is the single relaxation time. It should be noted that we append a compensation function $g_{k}\left(x,t\right)$ to recover Equation (1) exactly. There are some other important parameters in Equation (2), such as Δt representing the time step, Δx representing the lattice space step, $\alpha_{k}$ representing the discrete velocity. In this paper, we apply the simple and efficient D1Q5 velocity model. It means that the n=5 and $\alpha_{k}=ce_{k}=c\left\{0,1,-1,2,-2\right\}=\left\{0,c,-c,2c,-2c\right\}$, where c is a scale factor constant satisfying cΔt=Δx.

We let

(3) $$\frac{\tau_{0}}{\Delta t}=\tau ,\;i.e.,\;\frac{\Delta t}{\tau_{0}}=\frac{1}{\tau }.$$

Applying Taylor expansion to Equation (2) to $O(\Delta t^{5})$, and using Equations (2) and (3), we obtain

(4) $$\begin{array}{cc} \; & \Delta t\left(\partial_{t}+\alpha_{k}\partial_{x}\right)f_{k}+\frac{\Delta t^{2}}{2}{\left(\partial_{t}+\alpha_{k}\partial_{x}\right)}^{2}f_{k}+\frac{\Delta t^{3}}{6}{\left(\partial_{t}+\alpha_{k}\partial_{x}\right)}^{3}f_{k}+\frac{\Delta t^{4}}{24}{\left(\partial_{t}+\alpha_{k}\partial_{x}\right)}^{4}f_{k}\\ \; & +O\left(\Delta t^{5}\right)=\frac{f_{k}^{eq}-f_{k}}{\tau }+\Delta tg_{k}. \end{array}$$

We apply Chapman-Enskog(C-E) expansion to Equation (4) in space and time [15]:

(5) $$\partial_{x_{1}}=\frac{1}{\varepsilon }\partial_{x},$$

(6) $$\partial_{t}=\sum_{i=1}^{4}\varepsilon^{i}\partial_{t_{i}}+O\left(\varepsilon^{5}\right),$$

(7) $$f_{k}=\sum_{i=0}^{4}\varepsilon^{i}f_{k}^{i}+O\left(\varepsilon^{5}\right),$$

(8) $$g_{k}^{1}=\frac{1}{\varepsilon }g_{k}.$$

Substituting Equations (5)–(8) into Equation (4), we derive the equations from $\varepsilon^{0}$ to $\varepsilon^{4}$:

(9) $$\begin{array}{cc} \; & O\left(\varepsilon^{0}\right):\;-\frac{1}{\tau }\left(f_{k}^{0}-f_{k}^{eq}\right)=0,\;i.e.,\;\;\;f_{k}^{0}=f_{k}^{eq}, \end{array}$$

(10) $$\begin{array}{cc} \; & O\left(\varepsilon^{1}\right):\;\Delta t\partial_{t_{1}}f_{k}^{0}+\Delta t\alpha_{k}\partial_{x_{1}}f_{k}^{0}=-\frac{1}{\tau }f_{k}^{1}+\Delta tg_{k}^{1}, \end{array}$$

(11) $$O\left(\varepsilon^{2}\right):\;\Delta t\left(\partial_{t_{1}}f_{k}^{1}+\partial_{t_{2}}f_{k}^{0}+\alpha_{k}\partial_{x_{1}}f_{k}^{1}\right)+\frac{\Delta t^{2}}{2}\left(\partial_{t_{1}}^{2}+2\alpha_{k}\partial_{x_{1}}\partial_{t_{1}}+\alpha_{k}^{2}\partial_{x_{1}}^{2}\right)f_{k}^{0}=-\frac{1}{\tau }f_{k}^{2},$$

(12) $$\begin{array}{cc} \; & O\left(\varepsilon^{3}\right):\;\Delta t\left(\partial_{t_{1}}f_{k}^{2}+\partial_{t_{2}}f_{k}^{1}+\partial_{t_{3}}f_{k}^{0}+\alpha_{k}\partial_{x_{1}}f_{k}^{2}\right)+\Delta t^{2}\left(\partial_{t_{1},t_{2}}^{2}+\alpha_{k}\partial_{x_{1}}\partial_{t_{2}}\right)f_{k}^{0}+\frac{\Delta t^{2}}{2}\left(\partial_{t_{1}}^{2}\right.\\ \; & \left.+2\alpha_{k}\partial_{x_{1}}\partial_{t_{1}}+\alpha_{k}^{2}\partial_{x_{1}}^{2}\right)f_{k}^{1}+\frac{\Delta t^{3}}{6}\left(\partial_{t_{1}}^{3}+3\alpha_{k}\partial_{x_{1}}\partial_{t_{1}}^{2}+3\alpha_{k}^{2}\partial_{x_{1}}^{2}\partial_{t_{1}}+\alpha_{k}^{3}\partial_{x_{1}}^{3}\right)f_{k}^{0}=-\frac{1}{\tau }f_{k}^{3}, \end{array}$$

(13) $$\begin{array}{cc} \; & O\left(\varepsilon^{4}\right):\;\Delta t\left(\partial_{t_{1}}f_{k}^{3}+\partial_{t_{2}}f_{k}^{2}+\partial_{t_{3}}f_{k}^{1}+\partial_{t_{4}}f_{k}^{0}+\alpha_{k}\partial_{x_{1}}f_{k}^{3}\right)+\frac{\Delta t^{2}}{2}\left(2\partial_{t_{1},t_{3}}^{2}+\partial_{t_{2}}^{2}+2\alpha_{k}\partial_{x_{1}}\partial_{t_{3}}\right)f_{k}^{0}\\ \; & +\Delta t^{2}\left(\partial_{t_{1},t_{2}}^{2}+\alpha_{k}\partial_{x_{1}}\partial_{t_{2}}\right)f_{k}^{1}+\frac{\Delta t^{2}}{2}\left(\partial_{t_{1}}^{2}+2\alpha_{k}\partial_{x_{1}}\partial_{t_{1}}+\alpha_{k}^{2}\partial_{x_{1}}^{2}\right)f_{k}^{2}+\frac{\Delta t^{3}}{6}\left(3\partial_{t_{1}}^{2}\partial_{t_{2}}+6\alpha_{k}\partial_{x_{1}}\partial_{t_{1},t_{2}}^{2}\right.\\ \; & \left.+3\alpha_{k}^{2}\partial_{x_{1}}^{2}\partial_{t_{2}}\right)f_{k}^{0}+\frac{\Delta t^{3}}{6}\left(\partial_{t_{1}}^{3}+3\alpha_{k}\partial_{x_{1}}\partial_{t_{1}}^{2}+3\alpha_{k}^{2}\partial_{x_{1}}^{2}\partial_{t_{1}}+\alpha_{k}^{3}\partial_{x_{1}}^{3}\right)f_{k}^{1}+\frac{\Delta t^{4}}{24}\left(\partial_{t_{1}}^{4}+4\alpha_{k}\partial_{x_{1}}\partial_{t_{1}}^{3}\right.\\ \; & \left.+6\alpha_{k}^{2}\partial_{x_{1}}^{2}\partial_{t_{1}}^{2}+4\alpha_{k}^{3}\partial_{x_{1}}^{3}\partial_{t_{1}}+\alpha_{k}^{4}\partial_{x_{1}}^{4}\right)f_{k}^{0}=-\frac{1}{\tau }f_{k}^{4}. \end{array}$$

Using Equations (9)–(13), we obtain:

(14) $$\begin{array}{cc} \; & -\frac{f_{k}^{1}}{\tau \Delta t}=\left(\partial_{t_{1}}+\alpha_{k}\partial_{x_{1}}\right)f_{k}^{0}-g_{k}^{1}, \end{array}$$

(15) $$-\frac{f_{k}^{2}}{\tau \Delta t}=\left(\frac{\Delta t}{2}\partial_{t_{1}}^{2}+\Delta t\alpha_{k}\partial_{x_{1}}\partial_{t_{1}}+\frac{\Delta t}{2}\alpha_{k}^{2}\partial_{x_{1}}^{2}+\partial_{t_{2}}\right)f_{k}^{0}+\left(\partial_{t_{1}}+\alpha_{k}\partial_{x_{1}}\right)f_{k}^{1},$$

(16) $$\begin{array}{cc} \; & -\frac{f_{k}^{3}}{\tau \Delta t}=\left(\partial_{t_{3}}+\Delta t\partial_{t_{1},t_{2}}^{2}+\Delta t\alpha_{k}\partial_{x_{1}}\partial_{t_{2}}+\frac{\Delta t^{2}}{6}\partial_{t_{1}}^{3}+\frac{\Delta t^{2}}{2}\alpha_{k}\partial_{x_{1}}\partial_{t_{1}}^{2}+\frac{\Delta t^{2}}{2}\alpha_{k}^{2}\partial_{x_{1}}^{2}\partial_{t_{1}}+\frac{\Delta t^{2}}{6}\alpha_{k}^{3}\partial_{x_{1}}^{3}\right)f_{k}^{0}+\left(\partial_{t_{2}}+\frac{\Delta t}{2}\partial_{t_{1}}^{2}\right.\\ \; & \left.+\Delta t\alpha_{k}\partial_{x_{1}}\partial_{t_{1}}+\frac{\Delta t}{2}\alpha_{k}^{2}\partial_{x_{1}}^{2}\right)f_{k}^{1}+\left(\partial_{t_{1}}+\alpha_{k}\partial_{x_{1}}\right)f_{k}^{2}, \end{array}$$

(17) $$\begin{array}{cc} \; & -\frac{f_{k}^{4}}{\tau \Delta t}=\left(\partial_{t_{4}}+\Delta t\partial_{t_{1},t_{3}}^{2}+\frac{\Delta t}{2}\partial_{t_{2}}^{2}+\Delta t\alpha_{k}\partial_{x_{1}}\partial_{t_{3}}+\frac{\Delta t^{2}}{2}\partial_{t_{1}}^{2}\partial_{t_{2}}+\Delta t^{2}\alpha_{k}\partial_{x_{1}}\partial_{t_{1},t_{2}}^{2}+\frac{\Delta t^{2}}{2}\alpha_{k}^{2}\partial_{x_{1}}^{2}\partial_{t_{2}}+\frac{\Delta t^{3}}{24}\partial_{t_{1}}^{4}+\frac{\Delta t^{3}}{6}\alpha_{k}\partial_{x_{1}}\partial_{t_{1}}^{3}\right.\\ \; & \left.+\frac{\Delta t^{3}}{4}\alpha_{k}^{2}\partial_{x_{1}}^{2}\partial_{t_{1}}^{2}+\frac{\Delta t^{3}}{6}\alpha_{k}^{3}\partial_{x_{1}}^{3}\partial_{t_{1}}+\frac{\Delta t^{3}}{24}\alpha_{k}^{4}\partial_{x_{1}}^{4}\right)f_{k}^{0}+\left(\partial_{t_{3}}+\Delta t\partial_{t_{1},t_{2}}^{2}+\Delta t\alpha_{k}\partial_{x_{1}}\partial_{t_{2}}+\frac{\Delta t^{2}}{6}\partial_{t_{1}}^{3}+\frac{\Delta t^{2}}{2}\alpha_{k}\partial_{x_{1}}\partial_{t_{1}}^{2}\right.\\ \; & \left.+\frac{\Delta t^{2}}{2}\alpha_{k}^{2}\partial_{x_{1}}^{2}\partial_{t_{1}}+\frac{\Delta t^{2}}{6}\alpha_{k}^{3}\partial_{x_{1}}^{3}\right)f_{k}^{1}+\left(\partial_{t_{2}}+\frac{\Delta t}{2}\partial_{t_{1}}^{2}+\Delta t\alpha_{k}\partial_{x_{1}}\partial_{t_{1}}+\frac{\Delta t}{2}\alpha_{k}^{2}\partial_{x_{1}}^{2}\right)f_{k}^{2}+\left(\partial_{t_{1}}+\alpha_{k}\partial_{x_{1}}\right)f_{k}^{3}. \end{array}$$

Simplifying Equation (14), we have:

(18) $$f_{k}^{1}=-\tau \Delta t\left[\left(\partial_{t_{1}}+\alpha_{k}\partial_{x_{1}}\right)f_{k}^{0}-g_{k}^{1}\right].$$

Coupling Equations (15) and (18), one can obtain:

(19) $$f_{k}^{2}=-\tau \Delta t^{2}\left\{\left[\left(\frac{1}{2}-\tau \right)\partial_{t_{1}}^{2}+\left(\frac{1}{2}-\tau \right)\alpha_{k}^{2}\partial_{x_{1}}^{2}+\left(1-2\tau \right)\alpha_{k}\partial_{x_{1}}\partial_{t_{1}}+\frac{1}{\Delta t}\partial_{t_{2}}\right]f_{k}^{0}+\left(\tau \partial_{t_{1}}+\tau \alpha_{k}\partial_{x_{1}}\right)g_{k}^{1}\right\}.$$

Substituting Equations (18) and (19) into Equation (16), we have:

(20) $$\begin{array}{cc} f_{k}^{3}= & -\tau \Delta t^{3}\left\{\left[\frac{1}{\Delta t^{2}}\partial_{t_{3}}+\frac{1}{\Delta t}\left(1-2\tau \right)\partial_{t_{1},t_{2}}^{2}+\frac{1}{\Delta t}\left(1-2\tau \right)\alpha_{k}\partial_{x_{1}}\partial_{t_{2}}+\tau_{1}\partial_{t_{1}}^{3}+\tau_{2}\alpha_{k}\partial_{x_{1}}\partial_{t_{1}}^{2}+\tau_{2}\alpha_{k}^{2}\partial_{x_{1}}^{2}\partial_{t_{1}}\right.\right.\\ \; & \left.\left.+\tau_{1}\alpha_{k}^{3}\partial_{x_{1}}^{3}\right]f_{k}^{0}+\left[\frac{\tau }{\Delta t}\partial_{t_{2}}+\left(\frac{\tau }{2}-\tau^{2}\right)\partial_{t_{1}}^{2}+\left(\tau -2\tau^{2}\right)\alpha_{k}\partial_{x_{1}}\partial_{t_{1}}+\left(\frac{\tau }{2}-\tau^{2}\right)\alpha_{k}^{2}\partial_{x_{1}}^{2}\right]g_{k}^{1}\right\}. \end{array}$$

Finally, with the help of Equations (18)–(20), we rewrite Equation (17):

(21) $$\begin{array}{cc} f_{k}^{4}= & -\tau \Delta t^{4}\left\{\left[\frac{1}{\Delta t^{3}}\partial_{t_{4}}+\frac{1}{2\Delta t^{2}}\left(1-2\tau \right)\partial_{t_{2}}^{2}+\frac{1}{\Delta t^{2}}\left(1-2\tau \right)\partial_{t_{1},t_{3}}^{2}+\frac{1}{\Delta t^{2}}\left(1-2\tau \right)\alpha_{k}\partial_{x_{1}}\partial_{t_{3}}+\frac{\tau_{2}}{\Delta t}\partial_{t_{2}}\partial_{t_{1}}^{2}\right.\right.\\ \; & \left.\left.+\frac{6\tau_{1}}{\Delta t}\alpha_{k}\partial_{x_{1}}\partial_{t_{1},t_{2}}^{2}+\frac{\tau_{2}}{\Delta t}\alpha_{k}^{2}\partial_{x_{1}}^{2}\partial_{t_{2}}+\tau_{3}\partial_{t_{1}}^{4}+4\tau_{3}\alpha_{k}\partial_{x_{1}}\partial_{t_{1}}^{3}+6\tau_{3}\alpha_{k}^{2}\partial_{x_{1}}^{2}\partial_{t_{1}}^{2}+\tau_{3}\alpha_{k}^{4}\partial_{x_{1}}^{4}+4\tau_{3}\alpha_{k}^{3}\partial_{x_{1}}^{3}\partial_{t_{1}}\right]f_{k}^{0}\right.\\ \; & \left.+\left[\frac{\tau }{\Delta t^{2}}\partial_{t_{3}}+\frac{1}{\Delta t}\left(\tau -2\tau^{2}\right)\partial_{t_{1},t_{2}}^{2}+\frac{1}{\Delta t}\left(\tau -2\tau^{2}\right)\alpha_{k}\partial_{x_{1}}\partial_{t_{2}}+\tau_{4}\partial_{t_{1}}^{3}+3\tau_{4}\alpha_{k}\partial_{x_{1}}\partial_{t_{1}}^{2}+3\tau_{4}\alpha_{k}^{2}\partial_{x_{1}}^{2}\partial_{t_{1}}\right.\right.\\ \; & \left.\left.+\tau_{4}\alpha_{k}^{3}\partial_{x_{1}}^{3}\right]g_{k}^{1}\right\}. \end{array}$$

where:

$$\begin{array}{cc} \; & \tau_{1}=\tau^{2}-\tau +\frac{1}{6},\\ \; & \tau_{2}=3\tau^{2}-3\tau +\frac{1}{2},\\ \; & \tau_{3}=-\tau^{3}+\frac{3}{2}\tau^{2}-\frac{7}{12}\tau +\frac{1}{24},\\ \; & \tau_{4}=\tau^{3}-\tau^{2}+\frac{\tau }{6}. \end{array}$$

$f_{k}$ and $f_{k}^{eq}$ should satisfy the constraint:

(22) $$\sum_{k}f_{k}=\sum_{k}f_{k}^{eq}=u.$$

Using Equation (9), we obtain:

(23) $$\sum_{k}f_{k}^{0}=u,\;\sum_{k}f_{k}^{m}=0,\;m>0.$$

We can recover the equation exactly with minimal truncation error if both $f_{k}^{0}$ and $g_{k}$ satisfy the following conditions:    

(24) $$\begin{array}{cc} \; & \sum_{k}\alpha_{k}f_{k}^{0}=0, \end{array}$$

(25) $$\begin{array}{cc} \; & \sum_{k}\alpha_{k}^{2}f_{k}^{0}=0, \end{array}$$

(26) $$\begin{array}{cc} \; & \sum_{k}\alpha_{k}^{3}f_{k}^{0}=\frac{\gamma \left(t\right)P_{3}\left(u\right)}{\Delta t^{2}\tau_{1}}, \end{array}$$

(27) $$\begin{array}{cc} \; & \sum_{k}\alpha_{k}^{4}f_{k}^{0}=\frac{\eta \left(t\right)P_{4}\left(u\right)}{\Delta t^{3}\tau_{3}}. \end{array}$$

(28) $$\begin{array}{cc} \; & \sum_{k}g_{k}=0, \end{array}$$

(29) $$\begin{array}{cc} \; & \sum_{k}\alpha_{k}g_{k}=\frac{\alpha \left(t\right)p_{1}\left(u\right)}{\Delta t\tau }, \end{array}$$

(30) $$\begin{array}{cc} \; & \sum_{k}\alpha_{k}^{2}g_{k}=\frac{\beta \left(t\right)p_{2}\left(u\right)}{\Delta t^{2}\left(\frac{\tau }{2}-\tau^{2}\right)}, \end{array}$$

(31) $$\begin{array}{cc} \; & \sum_{k}\alpha_{k}^{3}g_{k}=0. \end{array}$$

Summing Equation (18) over *k*, and using Equations (22) and (23), we have:

(32) $$\partial_{t_{1}}u=0.$$

Summing Equation (19) over *k* and applying Equations (23)–(29), one can obtain:

(33) $$\partial_{t_{2}}u=-\frac{1}{\varepsilon^{2}}\partial_{x}\left[\alpha \left(t\right)p_{1}\left(u\right)\right].$$

Summing Equation (20) over *k* and with the aim of Equations (23)–(30), we obtain:

(34) $$\partial_{t_{3}}u=-\frac{1}{\varepsilon^{3}}\partial_{x}^{3}\left[\gamma \left(t\right)P_{3}\left(u\right)\right]-\frac{1}{\varepsilon^{3}}\partial_{x}^{2}\left[\beta \left(t\right)p_{2}\left(u\right)\right]-\frac{\Delta t}{\varepsilon^{2}}\left(1-2\tau \right)\partial_{x}\left[p_{1}\left(u\right)\right]\partial_{t_{1}}\left[\alpha (t)\right].$$

Summing Equation (21) over *k* and coupling with Equations (23)–(31), we have:

(35) $$\begin{array}{cc} \partial_{t_{4}}u= & \left(\tau \Delta t-\frac{\Delta t}{2}\right)\partial_{t_{2}}^{2}u-\frac{1}{\varepsilon^{4}}\partial_{x}^{4}\left[\eta \left(t\right)P_{4}\left(u\right)\right]-\frac{4\Delta t\tau_{3}}{\tau_{1}\varepsilon^{3}}\partial_{x}^{3}\left[P_{3}\left(u\right)\right]\partial_{t_{1}}\left[\gamma (t)\right]+\frac{\Delta t}{\varepsilon^{2}}\left(2\tau -1\right)\partial_{x}\left[p_{1}\left(u\right)\right]\partial_{t_{2}}\left[\alpha (t)\right]\\ \; & -\frac{\Delta t^{2}}{\varepsilon^{2}}\tau_{2}\partial_{x}\left[p_{1}\left(u\right)\right]\partial_{t_{1}}^{2}\left[\alpha (t)\right]-\frac{6\Delta t\tau_{1}}{\varepsilon^{3}\left(1-2\tau \right)}\partial_{x}^{2}\left[p_{2}\left(u\right)\right]\partial_{t_{1}}\left[\beta (t)\right]. \end{array}$$

Combining Equations (32)–(35) in order of $\varepsilon^{k}\left(k=1,2,3,4\right)$ and assuming ε=Δt, we are able to recover fourth order PDEs with TDVCs:

(36) $$\frac{\partial u}{\partial t}+\alpha \left(t\right)\frac{\partial }{\partial x}p_{1}\left(u\right)+\beta \left(t\right)\frac{\partial^{2}}{\partial x^{2}}p_{2}\left(u\right)+\gamma \left(t\right)\frac{\partial^{3}}{\partial x^{3}}p_{3}\left(u\right)+\eta \left(t\right)\frac{\partial^{4}}{\partial x^{4}}p_{4}\left(u\right)=\zeta ,$$

where the truncation error ζ of the model is:

$$\begin{array}{cc} \zeta = & -\varepsilon^{2}\left(1-2\tau \right)\partial_{x}\left[p_{1}\left(u\right)\right]\partial_{t_{1}}\left[\alpha (t)\right]-\varepsilon^{2}\frac{4\tau_{3}}{\tau_{1}}\partial_{x}^{3}\left[P_{3}\left(u\right)\right]\partial_{t_{1}}\left[\gamma (t)\right]-\varepsilon^{3}\left(1-2\tau \right)\partial_{x}\left[p_{1}\left(u\right)\right]\partial_{t_{2}}\left[\alpha (t)\right]\\ \; & -\varepsilon^{4}\tau_{2}\partial_{x}\left[p_{1}\left(u\right)\right]\partial_{t_{1}}^{2}\left[\alpha (t)\right]-\varepsilon^{2}\frac{6\tau_{1}}{1-2\tau }\partial_{x}^{2}\left[p_{2}\left(u\right)\right]\partial_{t_{1}}\left[\beta (t)\right]-\varepsilon^{5}\left(\frac{1}{2}-\tau \right)\partial_{t_{2}}^{2}u\\ \; & =O(\varepsilon^{2}). \end{array}$$

### 2.2. Selecting Appropriate Equilibrium Distribution Functions and Compensation Functions

By solving Equations (23)–(27), the equilibrium distribution functions $f_{k}^{0}\left(k=0,1,\ldots ,4\right)$ are expressed by

(37) $$\begin{array}{cc} \; & f_{0}^{0}=u+\frac{\eta \left(t\right)P_{4}\left(u\right)}{4c^{4}\Delta t^{3}\tau_{3}}, \end{array}$$

(38) $$\begin{array}{cc} \; & f_{1}^{0}=-\frac{\gamma \left(t\right)p_{3}\left(u\right)}{6c^{3}\Delta t^{2}\tau_{1}}-\frac{\eta \left(t\right)p_{4}\left(u\right)}{6c^{4}\Delta t^{3}\tau_{3}}, \end{array}$$

(39) $$\begin{array}{cc} \; & f_{2}^{0}=\frac{\gamma \left(t\right)p_{3}\left(u\right)}{6c^{3}\Delta t^{2}\tau_{1}}-\frac{\eta \left(t\right)p_{4}\left(u\right)}{6c^{4}\Delta t^{3}\tau_{3}}, \end{array}$$

(40) $$\begin{array}{cc} \; & f_{3}^{0}=\frac{\gamma \left(t\right)p_{3}\left(u\right)}{12c^{3}\Delta t^{2}\tau_{1}}+\frac{\eta \left(t\right)p_{4}\left(u\right)}{24c^{4}\Delta t^{3}\tau_{3}}, \end{array}$$

(41) $$\begin{array}{cc} \; & f_{4}^{0}=-\frac{\gamma \left(t\right)p_{3}\left(u\right)}{12c^{3}\Delta t^{2}\tau_{1}}+\frac{\eta \left(t\right)p_{4}\left(u\right)}{24c^{4}\Delta t^{3}\tau_{3}}.\; \end{array}$$

Then, assuming

(42) $$g_{4}=u^{2},$$

and solving Equations (28)–(31), the compensation functions $g_{k}\left(k=0,1,\ldots ,4\right)$ can be derived:

(43) $$\begin{array}{cc} \; & g_{0}=6u^{2}-\frac{\beta \left(t\right)p_{2}\left(u\right)}{c^{2}\Delta t^{2}\left(\frac{\tau }{2}-\tau^{2}\right)}-\frac{\alpha \left(t\right)p_{1}\left(u\right)}{2c\Delta t\tau }, \end{array}$$

(44) $$\begin{array}{cc} \; & g_{1}=-4u^{2}+\frac{\beta \left(t\right)p_{2}\left(u\right)}{2c^{2}\Delta t^{2}\left(\frac{\tau }{2}-\tau^{2}\right)}+\frac{\alpha \left(t\right)p_{1}\left(u\right)}{c\Delta t\tau }, \end{array}$$

(45) $$\begin{array}{cc} \; & g_{2}=-4u^{2}+\frac{\beta \left(t\right)p_{2}\left(u\right)}{2c^{2}\Delta t^{2}\left(\frac{\tau }{2}-\tau^{2}\right)}-\frac{\alpha \left(t\right)p_{1}\left(u\right)}{3c\Delta t\tau }, \end{array}$$

(46) $$\begin{array}{cc} \; & g_{3}=u^{2}-\frac{\alpha \left(t\right)p_{1}\left(u\right)}{6c\Delta t\tau }, \end{array}$$

(47) $$\begin{array}{cc} \; & g_{4}=u^{2}.\; \end{array}$$

## 3. Numerical Solutions

Our numerical simulation is based on Algorithm 1. Some fourth order PDEs with TDVCs are simulated to verify the effectiveness and stability of the model. For the treatment of boundary conditions, we use the nonequilibrium extrapolation method [16]. In addition, the initial and boundary value are determined by the exact solution. We apply global relative error (i.e., GRE) to measure error between the LBM solutions and the exact solutions.
**Algorithm 1:** Numerical SimulationSet initial value: $u_{0}$ = the exact solution, compute $p_{j}\left(u\right)\left(j=1,2,\ldots ,4\right)$ and $f_{k}\left(k=0,1,\ldots ,4\right)$$u=u_{0}$**for** r = 1 to Nt **do**    **for** i = 1 to m + 1 **do**        Compute $p_{j,i}\left(u\right)\left(j=1,2,\ldots ,4\right)$        Compute coefficients        Compute $f_{k,i}\left(u\right)$ and $g_{k,i}\left(u\right)\left(k=0,1,\ldots ,4\right)$        Collision    **end for**    Stream    Compute $u=\sum f_{k,i}^{0}\left(u\right)\left(k=0,1,\ldots ,4\right)$    Treat boundary condition**end for**

(48) $$\begin{array}{cc} \; & GRE=\frac{\sum_{n=1}^{L}\left|\vartheta \left(x_{n},t\right)-\vartheta^{{}^{\prime }}\left(x_{n},t\right)\right|}{\sum_{n=1}^{L}\left|\vartheta^{{}^{\prime }}\left(x_{n},t\right)\right|},\; \end{array}$$

where $\vartheta (x_{n},t)$ and $\vartheta^{\prime }\left(x_{n},t\right)$ are the LBM solution and the exact solution, respectively, *L* represents the number of lattices.

**Example** **1.** *Let $\alpha \left(t\right)=\frac{1}{2},\;p_{1}\left(u\right)=u^{2},\;\beta \left(t\right)=-1,\;p_{2}\left(u\right)=u,\;\gamma \left(t\right)=0,\;\eta \left(t\right)=1,\;p_{4}\left(u\right)=u$, and Equation (1) is transformed into Kuramoto-Sivashinsky (KS) equation with such a form:*

(49) $$\frac{\partial u}{\partial t}+u\frac{\partial u}{\partial x}-\frac{\partial^{2}u}{\partial x^{2}}+\frac{\partial^{4}u}{\partial x^{4}}=0.$$

We obtain the exact solution u(x,t) of Equation (49) by Ref. [1]

(50) $$\begin{array}{cc} u(x,t)= & b+\frac{15}{19\sqrt{19}}\left\{-3\tanh\left[k\left(x-bt-x_{0}\right)\right]+\tanh^{3}\left[k\left(x-bt-x_{0}\right)\right]\right\}. \end{array}$$

**Figure 1:**
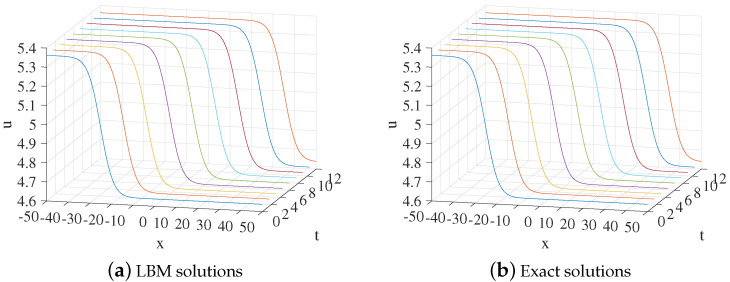
The 3D visual comparisons of LBM solutions (**a**) and exact solutions (**b**) from t=0 to t=12 for Example 1.

**Figure 2:**
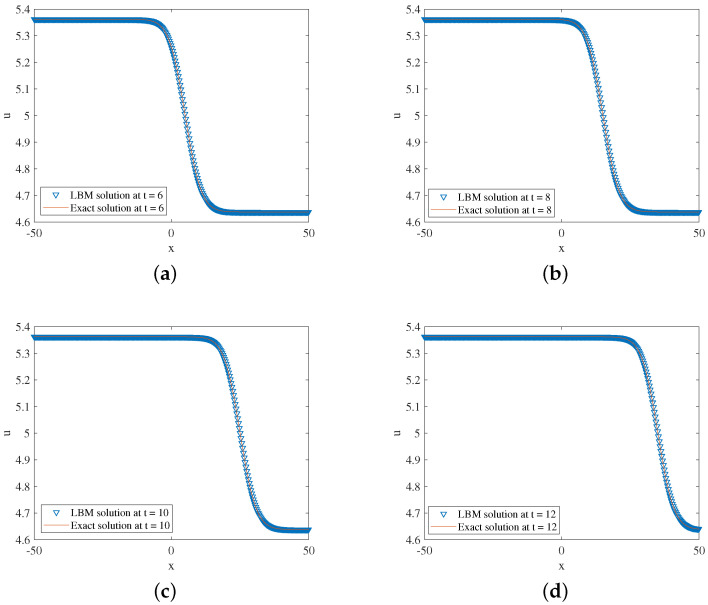
LBM solutions versus exact solutions at (**a**) t=6; (**b**) t=8; (**c**) t=10; (**d**) t=12 for Example 1.

In the simulation, we set x∈[−50,50], τ=1.28,Δx=0.1, Δt=0.0001. Some other parameters in Equation (50) are $k=\frac{1}{2\sqrt{19}},b=5$, and $x_{0}=-25$. The three-dimensional visual comparisons between the LBM solutions and exact solutions with time are shown in Figure 1. We show the space-time evolution graph of the LBM solutions and exact solutions in Figure 2. Besides, we compare the present model with the one in Ref. [9]. We also list the GRE of two models at different times in Table 1. It can find that our model is more accurate and performs better than the one in Ref. [9]. Based on Figure 1 and Figure 2 and Table 1, the LBM solutions and the exact solutions are in good agreement.

**Example** **2.** *When $\alpha \left(t\right)=\frac{1}{2},\;p_{1}\left(u\right)=u^{2},\;\beta \left(t\right)=1,\;p_{2}\left(u\right)=u,\;\gamma \left(t\right)=4,\;p_{3}\left(u\right)=u,\;\eta \left(t\right)=1,$$p_{4}\left(u\right)=u$, and Equation (1) is called the generalized Kuramoto-Sivashinsky (GKS) equation with the following form,*

(51) $$\frac{\partial u}{\partial t}+u\frac{\partial u}{\partial x}+\frac{\partial^{2}u}{\partial x^{2}}+4\frac{\partial^{3}u}{\partial x^{3}}+\frac{\partial^{4}u}{\partial x^{4}}=0.$$

We obtain the exact solution u(x,t) of Equation (51) by Ref. [2]

(52) $$\begin{array}{c} u\left(x,t\right)=b+9-15\left\{\tanh\left[k\left(x-bt-x_{0}\right)\right]+\tanh^{2}\left[k\left(x-bt-x_{0}\right)\right]-\tanh^{3}\left[k\left(x-bt-x_{0}\right)\right]\right\}. \end{array}$$

**Figure 3:**
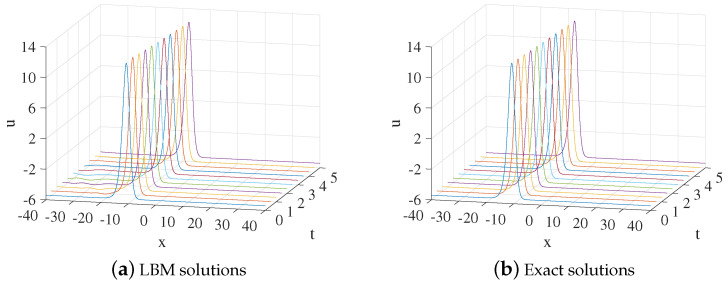
The 3D visual comparisons of LBM solutions (**a**) and exact solutions (**b**) from t=0 to t=5 for Example 2.

**Figure 4:**
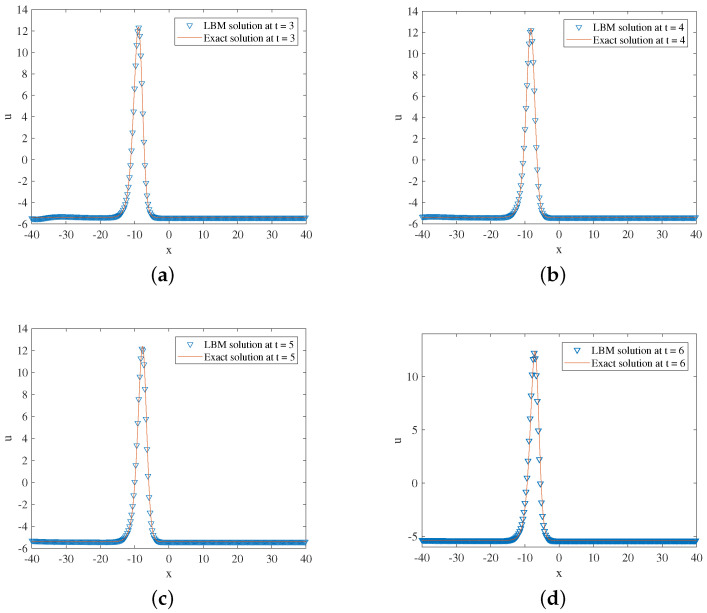
LBM solutions versus exact solutions at (**a**) t=3; (**b**) t=4; (**c**) t=5; (**d**) t=6 for Example 2.

In the simulation, we set x∈[−40,40], τ=1.3,Δx=0.1, and Δt=0.0001. Some other parameters in Equation (52) are $b=0.6,k=\frac{1}{2},$ and $x_{0}=-10$. The three-dimensional visual comparisons between the LBM solutions and exact solutions with time are shown in Figure 3. We show the space-time evolution graph of the LBM solutions and exact solutions in Figure 4. We also list the GRE of the model at different times in Table 2. Based on Figure 3 and Figure 4 and Table 2, the LBM solutions and the exact solutions are in good agreement.

**Example** **3.** *When $\alpha \left(t\right)=\frac{3}{4},\;p_{1}\left(u\right)=u^{4},\;\beta \left(t\right)=1,\;p_{2}\left(u\right)=u,\;\gamma \left(t\right)=-1,\;p_{3}\left(u\right)=u,\;$$\eta \left(t\right)\;=\;1,\;p_{4}\left(u\right)=u$, and Equation (1) is also a GKS equation with the different from:*

(53) $$\frac{\partial u}{\partial t}+3u^{3}\frac{\partial u}{\partial x}+\frac{\partial^{2}u}{\partial x^{2}}-\frac{\partial^{3}u}{\partial x^{3}}+\frac{\partial^{4}u}{\partial x^{4}}=0.$$

We obtain the exact solution u(x,t) of Equation (53) by Ref. [3]

(54) $$\begin{array}{c} u\left(x,t\right)=\frac{\sqrt{3}}{2\sqrt{2}}\tanh\left[\frac{\sqrt{3}}{4\sqrt{2}}\left(x-x_{0}-\frac{29}{144}t\right)+\frac{1}{2}\right]+\frac{1}{6}. \end{array}$$

**Figure 5:**
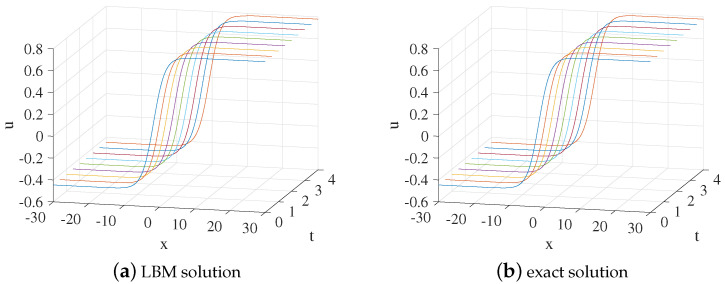
The 3D visual comparisons of LBM solutions (**a**) and exact solutions (**b**) from t=0 to t=4 for Example 3.

**Figure 6:**
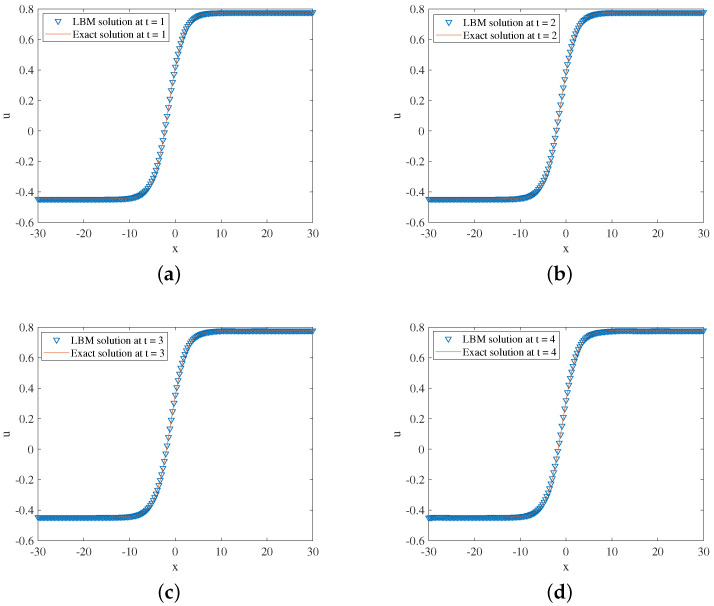
LBM solutions versus exact solutions at (**a**) t=1; (**b**) t=2; (**c**) t=3; (**d**) t=4 for Example 3.

In the simulation, we set x∈[−30,30], τ=1.8,Δx=0.1, and Δt=0.0001. One free parameter in Equation (54) is $x_{0}=0$. The three-dimensional visual comparisons between the LBM solutions and exact solutions with time are shown in Figure 5. We show the space-time evolution graph of the LBM solutions and exact solutions in Figure 6. We also list the GRE of the model at different times in Table 3. Based on Figure 5 and Figure 6 and Table 3, the LBM solutions and the exact solutions are in good agreement.

**Example** **4.** *Let $\alpha \left(t\right)=3\cos\left(2t\right),\;p_{1}\left(u\right)=u^{2},\;\beta \left(t\right)=0,\;\gamma \left(t\right)=\cos\left(2t\right),\;p_{3}\left(u\right)=u,\;\eta \left(t\right)=0$, and Equation (1) becomes a variable coefficient Korteweg-de Vries (KdV) equation with the following form,*

(55) $$\frac{\partial u}{\partial t}+6\cos\left(2t\right)u\frac{\partial u}{\partial x}+\cos\left(2t\right)\frac{\partial^{3}u}{\partial x^{3}}=0.$$

The exact solution [4] u(x,t) of Equation (55) is

(56) $$\begin{array}{c} u\left(x,t\right)=\frac{r}{2}{\mathrm{sech}}^{2}\left(\frac{\sqrt{r}}{2}\left(x-\frac{r}{2}\sin\left(2t\right)\right)-7\right). \end{array}$$

**Figure 7:**
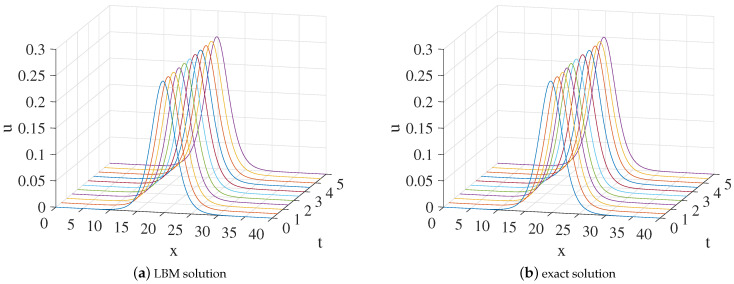
The 3D visual comparisons of LBM solutions (**a**) and exact solutions (**b**) from from t=0 to t=5 for Example 4.

**Figure 8:**
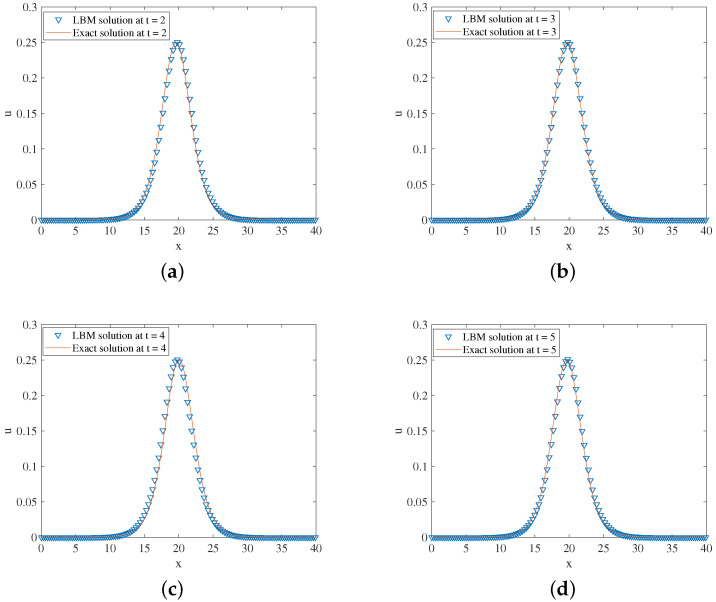
LBM solutions versus exact solutions at (**a**) t=2; (**b**) t=3; (**c**) t=4; (**d**) t=5 for Example 4.

In the simulation, we set x∈[0,40], τ=1.5,Δx=0.1, and Δt=0.0001. One free parameter *r* is set at 0.5 in Equation (56). The three-dimensional visual comparisons between the LBM solutions and exact solutions with time are shown in Figure 7. We show the space-time evolution graph of the LBM solutions and exact solutions in Figure 8. We also list the GRE of the model at different times in Table 4. Based on Figure 7 and Figure 8 and Table 4, the LBM solutions and the exact solutions are in good agreement.

## 4. Conclusions

In this paper, we developed a unified lattice Boltzmann model for the fourth order partial differential equation with time-dependent variable coefficients. In practice, as for the one-dimensional problems, we can use a unified lattice Boltzmann model to solve *n*th (n≤4 ) order PDEs with the specific form like Equations (1). From the C-E analysis, the macroscopic Equation (1) was recovered with second-order accuracy in time. Based on the proposed model and algorithm, some different types of PDEs such as in Equation (1), including the constant-coefficient KS equation, constant-coefficient GKS equation and variable-coefficient KdV equation, were numerically solved by selecting appropriate equilibrium distribution functions $f_{k}^{0}$, compensation functions $g_{k}$, the time step Δt, space step Δx and single relaxation time τ. The performance of the model was tested with a comparison between the numerical solution and the analytical solution. The numerical results show that the model is an effective method that can be used to simulate the equations such as Equatios (1).

Finally, we would like to point out that only one-dimensional problems with time-dependent variable coefficients in the form of Equation (1), were investigated in our model, while space-dependent variable coefficients PDEs and high-dimensional problems were not considered. We will replenish these inadequacies in the further work.

## Figures and Tables

**Table 1:** Comparison of LBM solutions and exact solutions for Example 1 at different times.

	t=6	t=8	t=10	t=12
GRE in Example 1	$4.1706\times 10^{-6}$	$5.2269\times 10^{-6}$	$6.1676\times 10^{-6}$	$7.2045\times 10^{-6}$
GRE in Ref. [9]	$7.8808\times 10^{-6}$	$9.5324\times 10^{-6}$	$1.0891\times 10^{-5}$	$1.1793\times 10^{-5}$

**Table 2:** Comparison of LBM solutions and exact solutions for Example 2 at different times.

	t=3	t=4	t=5	t=6
GRE	$7.012\times 10^{-3}$	$6.576\times 10^{-3}$	$1.082\times 10^{-2}$	$1.753\times 10^{-2}$

**Table 3:** Comparison of LBM solutions and exact solutions for Example 3 at different times.

	t=1	t=2	t=3	t=4
GRE	$2.3170\times 10^{-4}$	$4.7307\times 10^{-4}$	$7.3427\times 10^{-4}$	$1.127\times 10^{-3}$

**Table 4:** Comparison of LBM solutions and exact solutions for Example 4 at different times.

	t=2	t=3	t=4	t=5
GRE	$6.622\times 10^{-2}$	$2.628\times 10^{-2}$	$8.783\times 10^{-2}$	$4.597\times 10^{-2}$

## Data Availability

Not applicable.
